# Procedural justice, relative deprivation, and intra-team knowledge sharing: The moderating role of group identification

**DOI:** 10.3389/fpsyg.2023.994020

**Published:** 2023-02-21

**Authors:** Jin Wan, Mingyue Qin, Wenjun Zhou, Haiming Zhou, Pingping Li

**Affiliations:** ^1^School of Economics and Management, East China Jiaotong University, Nanchang, China; ^2^Jiangxi Institute of Talent and Industry Integration Development, East China Jiaotong University, Nanchang, China; ^3^Public Course Teaching Department, Shandong University of Science and Technology, Taian, China

**Keywords:** procedural justice, intra-team knowledge sharing, individual relative deprivation, group relative deprivation, group identification

## Abstract

How to promote employees’ knowledge-sharing behaviors has become a focus of managers and researchers. Based on the theory of relative deprivation, this study explored the mechanism of organizational procedural justice on employees’ intra-team knowledge sharing, as well as the mediating role of relative deprivation and the moderating role of group identification. A path analysis was conducted on 416 valid questionnaire data, and the results revealed that: (1) Procedural justice has a positive effect on intra-team knowledge sharing; (2) Both group relative deprivation and individual relative deprivation play a mediating role between procedural justice and intra-team knowledge sharing, but they have opposite effects. Procedural justice reduces both group relative deprivation and individual relative deprivation, but individual relative deprivation decreases employees’ intra-team knowledge sharing, while group relative deprivation increases it. (3) Group identification has an enhancing moderating effect on the relationship between group relative deprivation and intra-team knowledge sharing, while the moderating effect on the relationship between individual relative deprivation and intra-team knowledge sharing is not significant. Therefore, enterprises should make procedures such as performance appraisal and salary allocation justify and transparent to reduce individual relative deprivation, but should moderately trigger group relative deprivation flexibly according to the situation, while enhancing employee group identification through cultural construction.

## 1. Introduction

In a knowledge-based economy, the ability to compete among organizations is increasingly dependent on intangible assets such as employee expertise and experience skills within the organization (Zareie and Navimipour, 2016). Employees’ knowledge sharing as the exchange of information, experience, and expertise, is a key factor that affects competitive advantage and sustainable development of an organization (Rodboonsong and Sawasdee, 2020). However, knowledge sharing among employees is one of the major challenges facing organizational knowledge management today (Ullah et al., 2021; Virgilio, 2021).

Knowledge sharing is a fundamental behavior to create and apply knowledge in organizations (Castaneda and Ramirez, 2021), which is a kind of organizational citizenship behavior (Ficapal-Cusí et al., 2020). Constructive and mutually supportive relationships within organizations can accelerate the communication process and promote knowledge sharing among members, while distrustful behaviors and imbalance between giving and obtaining information within the organization threaten the effective sharing of tacit knowledge (Krogh, 1998).

Zigarmi et al. (2009) proposed a framework for employee work passion based on the social cognitive theory that organizational characteristics such as organizational justice, job characteristics such as autonomy, and individual characteristics such as motivation influence individuals’ behavior in organizational work through the role of individual cognition and emotion. Research has found that organizational justice positively affects employees’ knowledge sharing (Cugueró-Escofet et al., 2019). Colquitt et al. (2001) divided organizational justice into four dimensions: procedural, distributive, interpersonal, and informational, and found that the different dimensions of organizational justice do not have a consistent impact on employees’ attitudes and behaviors. And few studies have specifically examined the impact of different dimensions of organizational justice on intra-team knowledge sharing. Researchers have found that procedural justice can provide members with social–emotional needs (Murphy and Tyler, 2008), and more predict organizational system-referenced consequences such as organizational commitment, satisfaction, and negative emotions (Lucas, 2009; Lambert et al., 2010; Pignata et al., 2016). Whereas intra-team knowledge-sharing behavior is an organization-specific citizenship behavior (Ficapal-Cusí et al., 2020), this study focuses on the impact of procedural justice on intra-team knowledge sharing. Procedural justice has a direct positive impact on tacit knowledge sharing (Lin, 2007), it also can improve employees’ knowledge-sharing behavior through the mediation of organizational culture (Ibrahim et al., 2021). Therefore, procedural justice has a positive impact on intra-team knowledge-sharing behavior directly or indirectly.

In addition, relative deprivation theory states that relative deprivation is a link between the external environment and individual behavior, and individuals assess the external environment which they are in through social comparison, which leads to a certain degree of relative deprivation that affects their behavior (Smith and Ortiz, 2002). Research confirms that low-justice decision-making processes trigger feelings of relative deprivation in employees, leading to anger and obstructive behaviors, which, in turn, affect their cooperative intentions, job performance, and knowledge-sharing behaviors (Kim and Mauborgne, 1998; Melkonian et al., 2011). However, Tropp and Wright distinguished between individual relative deprivation and group relative deprivation and found that the two had different effects on employees’ attitudes and behaviors (Tropp and Wright, 1999). Thus, procedural justice may simultaneously have different effects on knowledge-sharing behavior through the mediation of individual relative deprivation and group relative deprivation. However, previous studies have rarely examined whether the two kinds of deprivation play different roles between procedural justice and knowledge-sharing behavior, which makes the understanding of the mechanism not comprehensive.

According to social identity theory, group identity is a part of an individual self-concept (Tajfel, 1978), and individual behavior is mutually driven by social identity or personal identity processes (Turner et al., 1989). Group cooperation and altruism are closely related to group identity (Tajfel, 1982). Knowledge-sharing behavior belongs to group behavior, and may also be influenced by group identification. Studies have concluded that there is a mutual causal effect between ingroup identification and relative deprivation (Zagefka et al., 2013). However, it has also been shown that group identification decreases individual relative deprivation and increases group relative deprivation (Smith et al., 1994; Kawakami and Dion, 1995; Ellemers, 2001), and that group identification moderates the relationship between relative deprivation and employees’ group behavior (Zhang et al., 2010). Group identification reduces the negative effects of negative emotions on employees’ organizational citizenship behaviors (Garcia-Prieto et al., 2007; Johnson et al., 2012; Carmeli et al., 2017), while relative deprivation is the subjective cognition and emotional experience of negative emotions such as anger and dissatisfaction generated by individuals (Xiong and Ye, 2016). Therefore, group identification may also moderate the relationship between relative deprivation and intra-team knowledge sharing. However, previous studies have not examined whether group identification also plays a moderate role between relative deprivation and intra-team knowledge sharing, especially whether it has the same moderation between the two kinds of deprivation and intra-team knowledge-sharing behavior, which limits the mastery of the relationship boundary of procedural justice and intra-team knowledge sharing.

In summary, based on relative deprivation theory and social identity theory, this study established a model with relative deprivation as a mediating variable and group identification as a moderating variable to explore the influence of procedural justice on employees’ intra-team knowledge sharing, to enrich the understanding of the influence mechanism of procedural justice on employees’ knowledge-sharing behavior, and provide a reference for organizations to improve corporate allocation procedures, enhance employees’ group identification, and promote employees’ intra-team knowledge sharing.

## 2. Theory and hypotheses

### 2.1. The impact of procedural justice on intra-team knowledge-sharing behavior

Procedural justice refers to employees’ perceived fairness about the processes and methods used in reward and other decisions (Thibaut and Walker, 1978), and it can directly affect many work-related attitudes and behaviors (Lind et al., 1990), such as positive job performance (Widyanti et al., 2020) and work engagement (Haynie et al., 2016). Researchers have found that procedural justice significantly enhances employees’ sense of organizational support, which promotes organizational citizenship behaviors (Zhang et al., 2017), yet employees who perceive low procedural justice express negative behaviors of refusing to work and deliberately destroying (Matteson et al., 2021). What is more, procedural justice not only directly affects organizational trust and organizational commitment (Widyanti et al., 2020; Ha and Lee, 2022), but also promotes employees’ active and voluntary organizational citizenship behavior in the workplace (Song et al., 2012; Chen and Jin, 2014). Intra-team knowledge-sharing behavior is a typical organizational citizenship behavior. Therefore, this study argues that employees will be more willing to share their knowledge, skills, and experience with other team members when they perceive that the organization’s allocation process is fair.

*Hypothesis 1*: Procedural justice has a positive impact on intra-team knowledge-sharing behavior.

### 2.2. The mediation of relative deprivation between procedural justice and intra-team knowledge sharing

Relative deprivation is the subjective cognition and emotional experience that individuals or groups perceive that they are at a disadvantage through horizontal or vertical comparison with the reference group, which leads to negative emotions such as anger and resentment (Xiong and Ye, 2016). It is a negative feeling produced by individuals after evaluating the injustices they have suffered (Grant and Brrown, 1995). Relative deprivation theory states that relative deprivation is a link between the external environment and individual behavior; individuals evaluate the external environment they are in through social comparison, and a certain degree of relative deprivation is generated when the external environment is unjust, which affects their behavior (Smith and Ortiz, 2002). Social justice has a negative effect on group members’ feelings of economic deprivation and social deprivation (Stouten et al., 2009; Smith et al., 2012), and organizational justice can reduce employees’ relative deprivation (Cole et al., 2010).

Tropp and Wright (1999) pointed out that a distinction needs to be made between group relative deprivation, which refers to the negative feelings of individuals who are dissatisfied with the experiences of the group to which they belong, and individual relative deprivation, which refers to the negative feelings of individuals who are dissatisfied with their own experiences, and the psychological and behavioral effects of the two relative deprivations are not consistent. When group members perceive distributional injustice, they will have a sense of individual relative deprivation (Hyunji et al., 2018), which can lead to employees feeling marginalized and even socially excluded (Anand et al., 2015), while group-based procedural injustice can lead to a sense of group deprivation (Smith et al., 2012; Osborne et al., 2015a).

*Hypothesis 2*: Procedural justice has a negative effect on individual relative deprivation.

*Hypothesis 3*: Procedural justice has a negative effect on group relative deprivation.

Because individual and group deprivation often occur simultaneously (Osborne et al., 2015b; Lilly et al., 2022), there are two possible relative deprivation effects between procedural justice and intra-team knowledge sharing. Individual relative deprivation emphasizes self-concern and group relative deprivation emphasizes group concern (Smith and Ortiz, 2002). The psychological and behavioral effects of the two on individuals are not consistent. Individual relative deprivation causes individuals to reduce prosocial behaviors (Zhang et al., 2016), implement negative workplace behaviors (Mishra and Novakowski, 2016), and even lead to anti-organizational behaviors (Kassab et al., 2020). Low-justice decision-making processes trigger a sense of relative deprivation in employees, leading to anger and obstructive behaviors, which, in turn, affect their collaborative intentions and knowledge-sharing behaviors (Kim and Mauborgne, 1998; Melkonian et al., 2011).

Therefore, this study argues that employees will be more inclined to reduce knowledge-sharing behaviors within their teams when they experience a sense of individual relative deprivation.

*Hypothesis 4*: Individual relative deprivation plays a mediating role between procedural justice and intra-team knowledge sharing. Specifically, through the mediation of individual relative deprivation, procedural justice has a positive impact on intra-team knowledge sharing.

Group relative deprivation is often associated with group-based responses and can predict collective action support (Lilly et al., 2022). Individuals who feel a sense of group deprivation are more inclined to engage in behaviors that support the group to which they belong (Krogh, 1998; Smith and Ortiz, 2002). As studies have confirmed, relative group deprivation promotes employees’ proactive change behavior (Kawakami and Dion, 1995) and motivates collective action to improve their group conditions by creating intergroup comparisons (Jetten et al., 2021). Intra-team knowledge sharing is typically a supportive behavior for the group. Accordingly, this study argues that when individuals experience group relative deprivation due to perceived injustice in their group, they will be more willing to work for the benefit of their group and more inclined to share the acquired knowledge and skills with other members of the team.

*Hypothesis 5*: Group relative deprivation mediates the relationship between procedural justice and intra-team knowledge sharing. Specifically, through the mediation of group relative deprivation, procedural justice has a negative impact on intra-team knowledge sharing.

### 2.3. The moderation of group identification between relative deprivations and intra-team knowledge sharing

Group identification means that an individual recognizes that he belongs to a specific social group, and also recognizes the emotional and value significance brought to him as a member of the group (Tajfel, 1978). It enhances members’ willingness to make their contributions for the collective benefit (Ben-Ner et al., 2009). When group identification is high, individuals’ motivation shifts from the individual level to the group level, enhancing cooperation with group members and motivating their behavior for the benefit of the group (Chen et al., 2007). Previous research confirms that group identification moderates the relationship between relative deprivation and employees’ group behaviors (Zhang et al., 2010). Group identification can reduce the negative effects of negative emotions on employees’ organizational citizenship behaviors (Garcia-Prieto et al., 2007; Johnson et al., 2012; Carmeli et al., 2017). Therefore, when employees with high group identification feel that they are deprived by the organization, they are less likely to reduce their team’s knowledge sharing for the benefit of the team.

*Hypothesis 6*: Group identification has a weakening effect on the relationship between individual relative deprivation and intra-team knowledge sharing. That is, the higher the individual’s group identification, the smaller the negative effect of individual relative deprivation on intra-team knowledge sharing.

Research confirmed that the higher the group member’s identification with the group, the more one can experience the negative emotions caused by relative deprivation (Xiong and Ye, 2016). Individuals with high ingroup identification more integrate group identification into their self-concept and experience more anger caused by relative deprivation, so they are more likely to act in favor of maintaining their group’s dominance (Pettit and Lount, 2011), such as engaging in cyber-cluster aggressive behavior (Song et al., 2018). Therefore, employees with high group identification will invest more energy in defending the interests of their group and may exhibit more intra-group knowledge sharing, when they are aware of group relative deprivation.

*Hypothesis 7*: Group identification plays an enhancing role in the relationship between group relative deprivation and intra-team knowledge sharing. That is, the higher the individual group identification, the greater the positive effect of group relative deprivation on intra-team knowledge sharing.

## 3. Research methods

### 3.1. Research participants

The participants were mainly knowledge-based employees in institutions, administrative organs, and various private enterprises in Hangzhou and other Chinese cities. Suitable participants were first selected relying on interpersonal resources, and then, the initial participants selected appropriate research objects in their organizations. Six hundred on-site paper questionnaires were distributed, 455 questionnaires were returned, and 416 of which were valid. Males accounted for 42.5% and females 57.5%; the average age was 41.03 years old, and the standard deviation was 8.149; 90.4% of subjects have a college or bachelor’s degree, and 6.7% have a master’s or doctoral degree.

The details of the sample data are shown in Table 1.

**Table 1:** Descriptive statistical analysis.

Characteristic	Categories	Proportion
Gender	Male	42.50%
	Female	57.50%
Education	Associate or bachelor’s degree	90.40%
	Master’s or doctoral degree	6.70%
Age	26–35 years old	19.60%
	36–45 years old	45.20%
	46–55 years old	27.80%
Position	Staff	78.80%
	First-line managers	4.30%
	Middle and top manager	16.90%
Workplace	Government-affiliated institutions.	66.60%
	Institutional units	23.50%
	All types of private enterprises	9.90%
Years of work	1–5 years	29.70%
	6–10 years	23.80%
	11–15 years	26.70%
	Over 16 years	19.80%
Staff number	More than 101 staff	63.30%
	61–100 staff	5.90%
	31–60 staff	21.40%
	30 staff and below	9.40%
City	Beijing, China	22.60%
	Hangzhou, China	26.20%
	Jinhua, China	18.30%
	Wenzhou, China	20.50%
	Ningbo, China	12.40%

### 3.2. Measures

Procedural justice was measured by the procedural justice dimension of the organizational justice scale developed by Colquitt et al. (2001), with seven items such as “In the process of developing policies for awarding compensation, those procedures have been free of bias.”

Individual relative deprivation was measured by a scale developed by Tropp and Wright (1999), with three items such as “I would say that I am worse off than others in our organization.”

Group relative deprivation was measured by a scale developed by Guimond and DubeSimard (1983), with two items such as “I feel that my department employees are worse off than other department employees.”

Intra-team knowledge sharing was measured by scale developed by Chow and Chan (2008), with five items such as “Sharing of my knowledge with team members is always an enjoyable experience.”

Group identification was measured by scale developed by Ashforth and Mael (1989), but the target was changed from the organization to the group, with six items such as “When someone praises my group, it feels like a personal compliment.”

All scales were scored on a five-point Likert scale, with 1–5 indicating “strongly disagree” to “strongly agree,” respectively. Control variables included gender, age, education, and length of service in the organization.

### 3.3. Statistical analysis

Spss25.0 was used for reliability test, common method bias test, correlation analysis and hierarchical regression analysis to test the reliability of the scales and the relationships between variables, with a p value smaller than 0.05 considered a statistically significant difference. And to further estimate the model, this study adopted the Bootstrap method with 5,000 samples in SPSS 25.0.

## 4. Results

### 4.1. Reliability and validity analysis

First, the reliability of scales was analyzed by SPSS25.0, and the Cronbach’s alpha of procedural justice, individual relative deprivation, group relative deprivation, knowledge sharing, and group identification scales was 0.872, 0.818, 0.872, 0.867, and 0.890, which showed scales selected had good reliability.

Confirmatory factor analysis was performed on all items of variables using MPLUS7.0 to test the discriminant validity between variables. This study compared the fit of models with one to five factors. The four factors model took individual relative deprivation and group relative deprivation as one factor; the three factors model took individual relative deprivation, group relative deprivation, and group identification as one factor; the two factors model took individual relative deprivation, group relative deprivation, group identification, and knowledge sharing as one factor. The fitting index (*X^2^/df* = 5.914, *CFI* = 0.797, *TLI* = 0.766, *SRMR* = 0.071, *RMESA* = 0.109) of the five-factor model was significantly better than that of the other models.

### 4.2. Common method bias test

Given that all data were filled in by participants at one time, Harman’s single-factor test was used, and it showed the percentage of variance explained by the first factor was 27.27%, which was lower than 40%. Furthermore, the method factor was added as a global factor on the basis of the five-factor model. The five-factor model fitted well (*X^2^/df* = 5.914, *CFI* = 0.797, *TLI* = 0.766, *SRMR* = 0.071, *RMESA* = 0.109), but the model could not fit adding the method factor. Both tests showed that there was no serious common method bias in this data.

### 4.3. Correlation analysis

Table 2 shows correlation coefficients of variables. Procedural justice is significantly positively correlated with intra-team knowledge sharing (*r* = 0.154, *p* < 0.05), and significantly negatively correlated with individual relative deprivation (*r* = −0.351, *p* < 0.001) and group relative deprivation (*r* = −0.338, *p* < 0.001); individual relative deprivation is significantly negatively correlated with intra-team knowledge sharing (*r* = −0.179, *p* < 0.001); and group relative deprivation is significant positive correlated with intra-team knowledge sharing (*r* = 0.139, *p* < 0.001). The hypotheses were initially tested.

**Table 2:** Descriptive statistics and correlations.

	Mean	SD	1	2	3	4	5	6	7	8	9
1. Gender	1.57	0.487									
2. Age	41.03	8.149	−0.132^**^								
3. Education	3.91	0.529	0.002	−0.425^***^							
4. Years of work	12.90	8.73	0.035	0.689^***^	−0.312^***^						
5. Procedural justice	3.15	0.790	−0.090	−0.076	−0.042	−0.183^***^					
6. Individual deprivation	2.61	0.793	−0.015	0.270^***^	−0.147^**^	0.351^***^	−0.351^***^				
7. Group deprivation	3.60	1.041	0.185^***^	0.338^***^	−0.226^***^	0.427^***^	−0.338^***^	0.466^***^			
8. Group identification	4.00	0.591	0.011	−0.031	−0.002	−0.140^**^	0.227^***^	−0.242^***^	0.085		
9. Knowledge sharing	4.13	0.529	0.041	−0.117^*^	0.043	0.006	0.154^**^	−0.179^***^	0.139^***^	0.288^***^	

*n* = 416; ^*^p < 0.05, ^**^p < 0. 01, ^***^p < 0.001; two-sided test.

### 4.4. Hypothesis test

#### 4.4.1. Hierarchical regression analysis

As shown in Table 3, the relationship between variables were tested using hierarchical regression analysis. In M5, only the control variables are added to the regression equation. To explore the relationship between procedural justice and intra-team knowledge sharing, based on M5, procedural justice was added to obtain M6. In M6, procedural justice has a significant direct positive effect on intra-team knowledge sharing (*β* = 0.155, *p* < 0.001). Hypothesis 1 was verified.

**Table 3:** Hierarchical regression analysis table.

Control variables	Individual deprivation				Group deprivation				Knowledge sharing							
	M1		M2		M3		M4		M5		M6		M7		M8	
	*β*	*SE*	*β*	*SE*	*β*	*SE*	*β*	*SE*	*β*	*SE*	*β*	*SE*	*β*	*SE*	*β*	*SE*
Gender	−0.031	0.075	−0.049	0.071	0.227***	0.080	0.212***	0.076	0.030	0.054	0.038	0.054	−0.061	0.051	−0.074	0.053
Age	0.030	0.007	0.048	0.006	−0.014	0.007	0.001	0.007	−0.209**	0.005	−0.217**	0.005	−0.206***	0.004	−0.220***	0.004
Education	−0.021	0.073	−0.073	0.070	−0.065	0.078	−0.108*	0.075	0.020	0.053	0.045	0.053	0.072	0.050	0.070	0.049
Years of work	0.297***	0.006	0.214***	0.005	0.384***	0.006	0.315***	0.006	0.154*	0.004	0.194**	0.004	0.116***	0.004	0.150*	0.004
Independent variable																
Procedural justice			−0.324***	0.044			−0.270***	0.047			0.155***	0.033	0.186***	0.033	0.133**	0.036
Mediate variable																
Individual deprivation													−0.248***	0.035	−0.225***	0.036
Group deprivation													0.414***	0.032	0.371***	0.033
Moderate variable																
Group identification															0.141**	0.038
Individual deprivation*																
Group identification															−0.110*	0.034
Group deprivation*																
Group identification															0.122*	0.031
R^2^	0.106		0.204		0.227		0.297		0.028		0.057		0.158		0.172	
F	12.605***		21.741***		30.113***		34.569***		2.970*		4.976***		10.938***		9.357***	
△R^2^	0.106		0.098		0.227		0.070		0.028		0.029		0.101		0.014	

*n* = 416; ^*^p < 0.05, ^**^p < 0. 01, ^***^p < 0.001; two-sided test.

In M1, only the control variables are added to the regression equation. To explore the relationship between procedural justice and individual relative deprivation, based on M1, procedural justice was added to obtain M2. In M2, procedural justice has a significant negative effect on individual relative deprivation (β = −0.324, *p* < 0.001), so hypothesis 2 was verified. In M3, only the control variables are added to the regression equation. To explore the relationship between procedural justice and group relative deprivation, based on M3, procedural justice was added to obtain M4. In M4, procedural justice has a significant negative effect on group relative deprivation (β = −0.270, *p* < 0.001), so hypothesis 3 was verified.

In addition, based on M6, individual relative deprivation and group relative deprivation were added to obtain M7. In M7, individual relative deprivation has a significant negative effect on intra-team knowledge sharing (β = −0.248, *p* < 0.001), and group relative deprivation has a significant positive effect on intra-team knowledge sharing (β = 0.414, *p* < 0.001). It indicated that individual deprivation and group deprivation mediate the relationship between organizational procedural justice and intra-team knowledge sharing. Hypotheses 4 and 5 were verified.

To explore the moderating effect of group identification,the interaction term of individual relative deprivation and group identification and group relative deprivation and group identification was constructed.On the basis of M7, the interaction term was added to obtain M8. In M8, both interaction terms have significant positive effects on intra-team knowledge sharing (β = −0.110, *p* < 0.05; β = 0.122, *p* < 0.05). It indicates that group identification moderates the relationship between individual relative deprivation and intra-team knowledge sharing, and group deprivation and intra-team knowledge sharing. Hypotheses 6 and 7 were verified.

#### 4.4.2. Bootstrap test

To further estimate the model, this study adopted the Bootstrap method with 5,000 samples in SPSS 25.0. According to different mediation paths, two model 14 tests are performed and results showed that procedural justice has a significant direct positive effect on intra-team knowledge sharing (β = 0.075 *p* < 0.032) and individual relative deprivation has a significant mediation between procedural justice and intra-team knowledge sharing (β = 0.045, CL = [0.022,0.067]). Hypothesis 4 was again tested. Also, the mediation group relative deprivation between procedural justice and intra-team knowledge sharing is significant (β = −0.053, CL = [−0.079,−0.032]). Hypothesis 5 was again verified. Thus, it showed that procedural justice not only directly and positively affects intra-team knowledge sharing, but also enhances it by reducing individual relative deprivation while also reduces it by reducing group deprivation.

In addition, the interaction between group relative deprivation and group identification has a significant positive effect on intra-team knowledge sharing (β = 0.111, *p* < 0.009), indicating that group identification plays a moderating role between group relative deprivation and intra-team knowledge sharing, but it does not moderate the relationship between individual relative deprivation and intra-team knowledge sharing. Hypothesis 7 was verified and hypothesis 6 was not verified.

**Figure 1:**
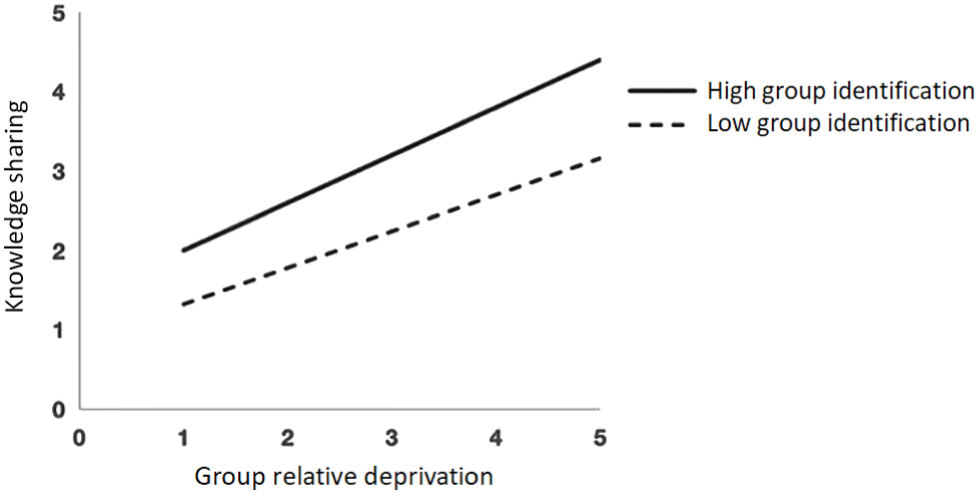
The moderating effect of group identification.

The moderating role of group identification between group relative deprivation and intra-team knowledge sharing was analyzed by simple slope analysis. The sample was divided into a high group identification group and a low group identification group based on the mean of group identification plus or minus one standard deviation. The moderating effect of group identification on the relationship between group relative deprivation and knowledge-sharing behavior is shown in Figure 1. Intra-team knowledge sharing of the high group identification group was higher than that of the low group identification group, and as the level of group relative deprivation increased, the intra-team knowledge sharing of the high group identification group became higher than that of the low group identification group, indicating that group identification played an enhancing moderating role in the positive effect of group relative deprivation on intra-team knowledge sharing.

## 5. Conclusion and discussion

### 5.1. Conclusion

This study aimed to explore the mechanisms of procedural justice on intra-team knowledge sharing, the mediating roles of individual and group relative deprivation and the moderating role of group identification based on relative deprivation theory. The study found that, overall, procedural justice has a positive effect on intra-team knowledge sharing. Both individual relative deprivation and group relative deprivation play a part mediation between procedural justice on knowledge sharing, but they act in opposite ways. When employees perceive deprivation against themselves in the organization, they reduce intra-team knowledge sharing, while when they perceive that their subgroups are subject to injustice organizational procedures, they increase knowledge sharing within the team. The higher the degree of individual identification with the group, the more they will stand in the perspective of the group and make knowledge-sharing behavior conducive to the group. However, the moderation of group identification between individual deprivation and intra-team knowledge sharing was weak and was not verified by 5,000 self-sampling tests.

### 5.2. Theoretical implications

First, procedural justice as an organizational factor affects employees’ intra-team knowledge sharing. Some studies have pointed out that procedural justice has a direct positive effect on tacit knowledge sharing (Lin, 2007). Previous research has found that procedural justice enhances employees’ knowledge sharing through the mediation of organizational culture (Ibrahim et al., 2021). According to the relative deprivation, this study found that procedural justice can also influence their intra-team knowledge sharing through relative deprivation, which reveals a new mechanism of procedural justice’s influence on knowledge sharing. It also validates the effectiveness of relative deprivation theory in explaining and predicting procedural justice and knowledge-sharing behavior, providing an empirical basis for relative deprivation theory.

Second, relative deprivation is a negative feeling resulting from the evaluation of injustice, but previous literature has pointed out that individual and group deprivation has different psychological and behavioral effects on individuals, and the two effects tend to be one positive and one negative. Individual relative deprivation can reduce prosocial behavior (Zhang et al., 2016) and even lead to anti-organizational behavior (Kassab et al., 2020), while group relative deprivation increases behaviors that support the group to which they belong (Krogh, 1998; Smith and Ortiz, 2002), promotes employee-initiated change behaviors (Kawakami and Dion, 1995), and motivates collective action (Jetten et al., 2021). In this study, by distinguishing individual relative deprivation from group relative deprivation, we found that procedural justice reduces group relative deprivation and individual relative deprivation, but individual relative deprivation decreases employees’ intra-team knowledge sharing, while group relative deprivation increases intra-team knowledge sharing, which also verifies the difference in the effects of the two relative deprivations. This also deepens the knowledge of the relationship between procedural justice and intra-team knowledge sharing, revealing its negative effects beyond the previously perceived positive effects of procedural justice on intra-team knowledge sharing.

Finally, based on social identity theory, this study found that group identification had an enhancing moderating effect on the relationship between group relative deprivation and intra-team knowledge sharing. Previous studies have found that the higher the degree of identification of group members with the group, the more they can experience the negative emotions brought about by group relative deprivation (Xiong and Ye, 2016). This study also found that group identification can enhance the impact of group deprivation on pro-group behavior. When employees with high group identification realize that their group is deprived, they will show more intra-team knowledge sharing to safeguard the interests of their group. However, group identification did not have a significant moderating effect on the relationship between individual relative deprivation and intra-team knowledge sharing. Perhaps because of the interaction between group identification and relative deprivation (Zagefka et al., 2013), individual deprivation makes employees feel that they are the only ones being discriminated against, making group identification weaker, and resulting in group identification losing its function of mitigating the negative effect of individual relative deprivation on intra-team knowledge-sharing behavior. Of course, this hypothesis needs further empirical testing.

### 5.3. Practice insights

The findings have the following implications for organizational management.

First, procedural justice has a significant positive effect on intra-team knowledge sharing. It has been found that procedural justice promotes employee performance (Widyanti et al., 2020), work engagement (Haynie et al., 2016), organizational citizenship behavior (Zhang et al., 2017), and improved job performance (Ha and Lee, 2022). This study also found that individuals are more willing to share their knowledge, skills, and experience to other members within the team when they perceive the process to be fair. Therefore, organizations should make efforts to improve procedural justice, specifically, they can standardize the management system, distinguish rewards and punishments, and make the company’s performance appraisal transparent; improve employee participation, involve employees in company decisions, and delegate employee power; share organizational resources, and encourage employees to develop themselves and communicate with each other. Organizations should try to make sure procedures are consistent for different personnel or at different times.

Secondly, procedural injustice has a positive influence on intra-team knowledge sharing through group relative deprivation instead. And a summary of relative deprivation studies by Xiong and Ye (2016) found that income gap, status gap, etc., can trigger group relative deprivation. Therefore, organizations can establish competition mechanisms among different departments and teams and set certain differences in distribution to appropriately trigger group deprivation and increase intra-team knowledge sharing.

Finally, triggering group relative deprivation is more effective for increasing knowledge sharing of employees with high group identification. Therefore, organizations should vigorously build a human-oriented organizational culture, and deepen communication and interaction between leaders and employees to improve employees’ organizational identification.

### 5.4. Limitations and prospects

The following limitations exist in this study. First, the data were self-reported and although the fitting index of the five-factor model was better than the other models, it was not good enough.

Although the statistical test showed no common method bias, follow-up study should try to use multiple data sources, such as paired sampling, to avoid common method bias, and collect at more types of organizations to test whether the conclusions of this study are stable in different types of organizations. Second, in this study, different variables were sampled at the same point. Therefore, future studies should collect data from multiple time points, and use a time-delay model to verify the causal relationships between the variables. Third, procedural justice in this study uses individual perceptions of procedural justice rather than organization level data. Some scholars pointed out that there is a moderate correlation between subjective perceptions and objective job characteristics (Algera, 1983). Therefore, it is reasonable to use the subjective perceptions of procedural justice to reflect organizational procedural justice. However, follow-up studies can also examine the model by taking procedural justice as an organizational level variable for cross-level research. Finally, this study only explored intra-team knowledge sharing, while the role of procedural justice on inter-departmental and inter-organizational knowledge sharing and their mechanisms needs to be further studied.

## Data availability statement

The raw data supporting the conclusions of this article will be made available by the authors, without undue reservation.

## Ethics statement

Ethical review and approval was not required for the study on human participants in accordance with the local legislation and institutional requirements. The patients/participants provided their written informed consent to participate in this study.

## Author contributions

JW: conceptualization, methodology, and revision. MQ: writing and translation. WZ: data analysis. HZ: design questionnaire, chart making, and table making. PL: questionnaire collection and reference collection. All authors contributed to the article and approved the submitted version.

## Funding

This research is supported by the National Natural Science Foundation of China (nos. 72161014 and 72162017) and the Social Science Foundation of Jiangxi Province (no. 22JY23).

## Conflict of interest

The authors declare that the research was conducted in the absence of any commercial or financial relationships that could be construed as a potential conflict of interest.

## Publisher’s note

All claims expressed in this article are solely those of the authors and do not necessarily represent those of their affiliated organizations, or those of the publisher, the editors and the reviewers. Any product that may be evaluated in this article, or claim that may be made by its manufacturer, is not guaranteed or endorsed by the publisher.
